# Month 2 Culture Status and Treatment Duration as Predictors of Tuberculosis Relapse Risk in a Meta-Regression Model

**DOI:** 10.1371/journal.pone.0071116

**Published:** 2013-08-05

**Authors:** Robert S. Wallis, Cunshan Wang, Daniel Meyer, Neal Thomas

**Affiliations:** Specialty Care, Pfizer, Groton, Connecticut, United States of America; National Institute of Allergy and Infectious Disease, United States of America

## Abstract

**Background:**

New drugs and regimens with the potential to transform tuberculosis treatment are presently in early stage clinical trials.

**Objective:**

The goal of the present study was to infer the required duration of these treatments.

**Method:**

A meta-regression model was developed to predict relapse risk using treatment duration and month 2 sputum culture positive rate as predictors, based on published historical data from 24 studies describing 58 regimens in 7793 patients. Regimens in which rifampin was administered for the first 2 months but not subsequently were excluded. The model treated study as a random effect.

**Results:**

The model predicted that new regimens of 4 or 5 months duration with rates of culture positivity after 2 months of 1% or 3%, would yield relapse rates of 4.0% or 4.1%, respectively. In both cases, the upper limit of the 2-sided 80% prediction interval for relapse for a hypothetical trial with 680 subjects per arm was <10%. Analysis using this model of published month 2 data for moxifloxacin-containing regimens indicated they would result in relapse rates similar to standard therapy only if administered for ≥5 months.

**Conclusions:**

This model is proposed to inform the required duration of treatment of new TB regimens, potentially hastening their accelerated approval by several years.

## Introduction

Modern tuberculosis chemotherapy has evolved as a result of hundreds of randomized, controlled clinical trials conducted over decades. Through the introduction of rifampin and pyrazinamide, these trials shortened the required duration of treatment from 18 months to the present 6 months without increasing the rate of recurrence due to relapse. TB treatment is presently poised for a second such transformation [1], with the development of several promising new drugs with entirely novel mechanisms of action. The early development of new regimens containing these compounds has been facilitated by a prior report qualifying month 2 sputum culture status as biomarker for relapse [2]. That study used meta-regression analysis to examine 30 pairs of regimens of equal duration, selected if the two treatments differed either uniformly throughout the treatment period or only during the first 2 months. The analysis found that changes in treatment that affected relapse risk were highly likely to also affect month 2 culture status (*P*<0.00001). The finding supported the use of month 2 culture status in phase 2 trials to select drug doses and evaluate new drug combinations. It did not directly inform the required duration of these new treatments, however, potentially impeding their accelerated or conditional approval for patients at greatest medical need, such as those with multiple or extensive drug resistance [1].

The present analysis extends that report by examining the relationship between 3 parameters –month 2 culture status, treatment duration, and relapse risk – with the goal of informing the required duration of treatment of new TB regimens for phase 3 trials and accelerated regulatory approvals.

## Methods

The medical literature was reviewed to identify randomized, prospective controlled trials of tuberculosis chemotherapy in patients with first episodes of sputum smear-positive pulmonary disease fully sensitive to the drugs included in the assigned treatments. The review process was consistent with PRISMA guidelines [3]. Studies conducted by the British Medical Research Council (MRC) were initially identified through examination of a review article [4]. Additional studies conducted by MRC, and those conducted by other research organizations were identified by searching Pubmed using the search terms “tuberculosis” and “clinical trial”. Articles with publication dates through January 2013 were included. Studies were included in this analysis if the published report included the number of subjects in each arm, the composition and total duration of treatment, sputum culture status after 2 months of treatment as determined using solid agar medium, and rate of relapse 1–2 years after treatment was completed. Regimens from these studies were excluded if rifampin was given for the initial 2 months but not subsequently. This was warranted because the full benefit of rifampin on relapse requires it be continued throughout treatment [5], [6]; if rifampin is discontinued at 2 months, relapse rates will exceed those anticipated by 2 month data.

Data were extracted from publications into a database by one author (RSW). Regimens were scored according to inclusion of rifampin and pyrazinamide. Those lacking both rifampin and pyrazinamide were scored as 0. Those in which both drugs were administered daily during the intensive phase and in which rifampin was given either daily or intermittently during the continuation phase were scored as 1. All other regimens including rifampin or pyrazinamide were scored as 0.5. Regimens were also scored according to the geographic region in which they were studied. Those studied in Africa were scored as 1; those studied in Asia were scored as 0. Global studies including Africa were scored as 0.5.

A meta-regression model was developed in which relapse rate was the dependent variable, and proportion of subjects with positive cultures at month 2, and treatment duration, the independent variables. Rates (month 2 culture positivity and relapse) were transformed using the logit function; regimens with reported rates of zero were assigned values of 0.005 (0.5%). Treatment duration was transformed using the natural log function. These transformations improved model fit. The model treated study as a random effect. The residual variance was assumed fixed, whereas the study variance was estimated by restricted maximum likelihood using the SAS MIXED procedure [7]. The residual variance was the variance of the logit transformed relapse rate, *i.e.,* 1/(*Np*(1-*p*)) where *N* was the sample size and *p* was the relapse rate of each arm. Regression parameters were estimated via weighted least squares using the inverse of the sum of the residual and study variance as the weight.

From the fitted model, we predicted relapse rates at given rates of month 2 culture positivity and treatment duration along with 2-sided 80% confidence intervals (CI). We also constructed 2-sided 80% prediction intervals (PI) for a hypothetical Phase 3 trial with 680 subjects per arm. The prediction error variance on the logit scale was *SE^2^+ Vs +1/(N_new_q*(1-*q*)), where *q* was the model predicted logit relapse rate at a given level of month 2 culture positive rate and treatment duration, *SE* was the standard error of *q*, *N_new_* was the number of subjects per arm of the hypothetical trial, and *Vs* was the estimated variance associated with the study. The intervals were formed on the logit scale and back-transformed to an ordinary percent scale. Diagnostic plots such as predicted value *vs*. standardized residual, histogram and Q-Q plot of the residuals were used to check model fit. Scatter plots of the data on both the original and transformed scales were examined. Additional models that included either region (classification variable with 3 levels), or interaction between logit transformed 2-month culture positive rate and natural log transformed treatment duration, as an added covariate, were fitted as sensitivity analyses. Lastly, a Bayesian random-effects logistic regression analysis assuming diffuse priors was conducted using OpenBugs [8], as a supportive analysis. That model did not require the normal approximations utilized by the mixed linear model.

## Results

Ninety two unique reports of regimens were initially identified that met the specified inclusion criteria. Of these, 34 were removed due to the inclusion of rifampin in the initial 2 months but not subsequently (Figure S1). The resulting dataset, representing 58 regimens from 24 trials, was remarkable for its size and diversity. The dataset is summarized in table 1 and described fully in Table S1. The 3 regimens lacking both rifampin and pyrazinamide were among the most diverse, with durations ranging from 6–18 months and relapse rates ranging from 4–32%. Across all regimens and geographic regions, logit transformed month 2 culture positivity was inversely correlated with inclusion of rifampin or/and pyrazinamide (RZ status), although a weak interaction was present between RZ status and geographic region (Figure S2). Two month cultures were least likely to be positive in studies conducted solely in Asia. These observations, which are consistent with other reports [9], [10], confirmed the basic integrity of the data set.

**Table 1:** Characteristics of included regimens.

Parameter	N or mean (range)
Subjects	7793
Studies	24
Regimens	58
Inclusion of rifampin and pyrazinamide(none/partial/full)	3/32/23
Geographic region (Asia/global/Africa)	42/3/13
Relapse rate	6.7% (0–29%)
Month 2 culture positivity rate	16.4% (1%–58%)
Duration of treatment	6.3 months (3–18)

**Figure 1:**
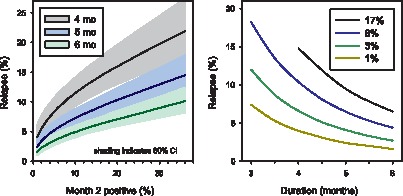
Two views of the interrelationship of relapse, duration, and month 2 culture status, as predicted by meta-regression analysis. Left: Relapse vs. month 2 culture positivity for regimens of 4, 5, and 6 months duration. Right: Relapse vs. duration for regimens with month 2 positive rates of 1 to 17%.

Parameter estimates on the logit scale predicting relapse risk based on month 2 culture status and duration are shown in table 2. Highly significant relationships were identified for both parameters (*P*<.0001). Diagnostic plots for this predictive model are shown in Figures S3, S4 and S5. The variance estimate associated with the study effect was 0.392; the variation explained by the model (R^2^) was 0.301. The predicted relapse rates and 80% confidence intervals for new regimens of various durations using this model are shown in figure 1. The model predicted that new regimens of 4 or 5 months duration, with month 2 culture positive rates of 1% or 3%, would yield relapse rates of 4.0% or 4.1%, respectively. We also calculated the 80% prediction intervals for a hypothetical trial with 680 subjects per arm. In both cases, the upper limits of the 2-sided 80% prediction interval for relapse for a hypothetical trial with 680 subjects per arm were <10% (table 3).

**Table 2:** Parameter estimates for meta-regression model.

Parameter	Estimate	SE	P
Intercept	2.1471	0.6092	0.0018
Natural log treatment duration	−2.2670	0.2958	<.0001
Logit month 2 culture positive rate	0.4756	0.1063	<.0001

**Table 3:** Predicted relapse rates for hypothetical regimens of 4, 5, and 6 months duration, according to the rate of sputum culture positivity at 2 months.

Treatment duration (mo)	Month 2 positive rate (%)	Predicted relapse rate (%)	80% CI	80% PI
4	1	4	2.6–6.1	1.6–9.7
4	2	5.5	3.9–7.7	2.3–12.5
4	3	6.6	4.9–8.9	2.8–14.6
4	4	7.5	5.7–9.8	3.3–16.3
4	5	8.3	6.5–10.7	3.7–17.8
4	6	9.1	7.2–11.5	4.1–19.1
4	8	10.4	8.3–12.8	4.7–21.3
4	10	11.5	9.3–14.1	5.3–23.3
4	13	13	10.6–15.9	6–25.9
4	18	15.2	12.4–18.6	7.2–29.5
4	26	18.3	14.8–22.6	8.7–34.5
4	36	21.9	17.3–27.4	10.6–39.9
5	1	2.4	1.6–3.8	0.9–6.2
5	2	3.4	2.4–4.8	1.4–8
5	3	4.1	3–5.5	1.7–9.4
5	4	4.7	3.6–6.1	2–10.6
5	5	5.2	4.1–6.6	2.2–11.6
5	6	5.7	4.5–7.1	2.5–12.5
5	8	6.5	5.3–8	2.9–14.1
5	10	7.3	6–8.8	3.2–15.5
5	13	8.3	6.9–9.9	3.7–17.3
5	18	9.8	8.2–11.6	4.5–20.1
5	26	11.9	9.8–14.4	5.5–24
5	36	14.5	11.6–17.9	6.7–28.4
6	1	1.6	1–2.6	0.6–4.3
6	2	2.3	1.6–3.2	0.9–5.6
6	3	2.7	2–3.8	1.1–6.6
6	4	3.1	2.4–4.2	1.3–7.4
6	5	3.5	2.7–4.5	1.5–8.1
6	6	3.8	3–4.9	1.6–8.7
6	8	4.4	3.6–5.4	1.9–9.9
6	10	4.9	4–6	2.2–10.9
6	13	5.6	4.7–6.7	2.5–12.3
6	18	6.7	5.6–8	3–14.3
6	26	8.2	6.8–9.9	3.7–17.3
6	36	10.1	8.2–12.4	4.6–20.8

The confidence interval (CI) indicates uncertainty regarding the population relapse rate. The prediction interval (PI) indicates uncertainty regarding the predicted relapse rate in a future study with 680 subjects per arm.

Sensitivity analyses including region, or interaction between logit transformed month 2 culture positive rate and natural log transformed treatment duration, as additional covariates, yielded similar results. Neither covariate reached statistical significance (*P*≥0.64), nor did either significantly affect model predictions from a clinical perspective. Lastly, a Bayesian random-effects logistic regression analysis assuming diffuse priors produced results that also were consistent with the primary model results (not shown).

The potential impact of this model on the design of phase 3 trials to shorten TB treatment from its present 6 months is illustrated in table 4. Five studies of 2 months duration have reported the effect on sputum culture status of adding moxifloxacin or substituting it for part of standard TB therapy [11]–[15]. These studies did not inform model design, as they did not report relapse as an endpoint. In addition, one trial did not randomly assign patients to treatments [11]; a second used both solid and liquid culture [15]. Nonetheless, four of these trials showed numerically superior culture rates at 2 months in the moxifloxacin arm (center columns) that could potentially indicate a role in shortening therapy. The relapse rates predicted by our model based on these data (right columns) indeed support a role of moxifloxacin to shorten treatment to 5 months; however, further shortening to 4 months was predicted to incur increased relapse risk. This conclusion is further supported by the recent report in abstract form of the results of the RIFAQUIN trial, in which the substitution of moxifloxacin for isoniazid reduced the proportion of subjects with positive cultures at 2 months from 14.6% to 9.6% [16]. Subjects in the experimental arms in that trial continued with moxifloxacin plus rifapentine either twice weekly for 2 additional months (4 months total duration) or once weekly for 4 additional months (6 months total duration). We calculated the relapse rate in subjects treated for a total of 4 months in that trial to be 14.1% and 14.7% in mITT and per protocol analyses, respectively. This was numerically similar to and within the limits predicted by the model (11.3%, 80% prediction interval: 5.0%–23.6%).

**Table 4:** Observed rates of culture positivity at month 2 (center columns) and predicted rates of relapse (right columns) based on data from five 2-month trials of experimental moxifloxacin-containing regimens.

Study	Month 2 positive rate		Predicted relapse rate		
	moxifloxacin	HRZE	moxifloxacin regimen		HRZE
	regimen		*4 months*	*5 months*	*6 months*
Wang [11]	8.0%	17.0%	10.4% (4.7–21.3)	6.5% (2.9–14.1)	6.5% (2.9–13.9)
Dorman [12]	8.5%	12.8%	10.7% (4.8–21.8)	6.7% (3–14.4)	5.6% (2.5–12.2)
Conde [13]	8.0%	26.0%	10.4% (4.7–21.3)	6.5% (2.9–14.1)	8.2% (3.7–17.3)
Rustomjee [14]	18.0%	36.0%	15.2% (7.2–29.5)	9.8% (4.5–20.1)	10.1% (4.6–20.8)
Burman [15]	29.0%	29.0%	19.4% (9.3–36.2)	12.7% (5.9–25.3)	8.8% (4–18.3)

Relapse rates were predicted using the equation parameters in table 2. Treatment durations of 4 and 5 months were considered for the moxifloxacin regimens; a duration of 6 months was assumed for standard (HRZE) treatment. Parentheses indicate 80% prediction intervals for a trial with 680 subjects per arm. Findings from 4 of the 5 studies could be considered as supporting a role for moxifloxacin in 5 month regimens; however, none supported a role as a 4 month regimen, due to increased relapse risk.

## Discussion

This study examined the inter-relationship of culture status after 2 months of treatment, total treatment duration, and relapse risk in historical data of patients entered in clinical trials of treatment of pulmonary tuberculosis. The resulting mathematical model predicted acceptable rates of relapse of new 4 and 5 month regimens with month 2 culture positivity rates of 1% and 3%, respectively. This model is proposed to inform the duration of treatment in future phase 3 trials. In addition to RIFAQUIN, two other large and costly trials of 4 month fluoroquinolone-containing regimens (REMox-TB and OFLOTUB) with relapse as the main endpoint are expected to report top line results in the coming year. It is unlikely that these trials would have been conducted in their present form had the model been available to guide their design.

This model may also guide treatment of patients with drug-resistant TB following accelerated approval of new regimens for which optimal duration is not yet known [1]. This may hasten by several years the availability of new regimens to patients with high unmet medical need, such as those with highly drug-resistant infections, in whom the balance between potential benefits and risks favors the acceptability of new regimens despite greater therapeutic uncertainty.

Limitations of this analysis must be acknowledged. The promise of biomarkers measured early during treatment lies in their capacity to predict later clinical events. This promise can only be realized if subsequent treatment continues as planned. Human and microbial populations may change over time, potentially affecting the validity of predictions based on historical data. The confidence and prediction intervals in this model are wide, making the model appropriate for the evaluation of regimens but not for guiding the treatment of individual patients. This likely indicates that other, unmeasured factors influence the outcome of TB treatment. The data from which the model was developed came from patients with first episodes of sputum smear positive pulmonary TB in patients with fully drug-susceptible disease. Patients with chronic disease who have failed multiple prior treatments may have more extensive lung involvement at baseline; they may as a result show delayed culture conversion and therefore require more prolonged treatment. A conservative, staged approach is therefore recommended in the translation of these findings to patients with chronic drug-resistant infections, particularly in the setting of accelerated approvals outside of the close monitoring afforded by traditional clinical trials. Extended treatment may also be considered for initial licensing of a new regimen in African countries where delayed sputum culture conversion has been observed. New drugs or other adjunctive treatments with novel mechanisms of action may be developed in the future that are administered only during the intensive phase of treatment. Additional data will be required to model the outcomes of such treatments. Newer culture methods using liquid medium show greater sensitivity for detection of *M. tuberculosis* in sputum, particularly in patients who have started treatment. Additional data using liquid culture will be required to model these responses. Progression of new infection has been increasingly recognized as a cause of recurrent tuberculosis after apparent cure, especially in HIV-infected persons. HIV status was known for only a small proportion of patients enrolled in these trials. TB relapse generally occurs within the first 6–12 months after the end of treatment [17]. In contrast, the risk of recurrence due to reinfection increases with local TB prevalence [18], and is unaffected by time since end of treatment [19], [20]. Although these observations can aid in the estimation of the influence of reinfection on recurrence, molecular strain typing will be required for accurate determination of the etiology of recurrent disease in individual patients. Lastly, the number of trials included in this model that are shorter than 6 months is relatively small. The model will require revision as additional data from larger studies become available.

In summary, models to predict relapse risk of new TB regimens based on month 2 culture status and treatment duration can guide the design of phase 3 trials and the accelerated treatment of patients with drug-resistant disease.

## Supporting Information

Figure S1 **Study flow chart according to PRISMA guidelines. From reference** [**3**] **.**
(PDF)

Figure S2 **Mean rates of month 2 culture positivity in regimens classified according to inclusion of rifampin and pyrazinamide, and according to geographic region.** Regimens were scored 0 if they included neither drug, and 1 if both drugs were given daily for the first 2 months, and if rifampin was given daily or intermittently thereafter. All other regimens that included either drug were scored 0.5. A weak interaction of the two parameters on culture status was observed.
(EPS)

Figure S3 **Scatter plot of logit 2-mo culture positive rates vs. logit relapse rates.**
(TIF)

Figure S4 **Scatter plot of natural log of treatment duration vs. logit relapse rates.**
(TIF)

Figure S5 **Diagnostic plots of standardized residuals for logit relapse rates.** A) Predicted values vs. residuals, B) Histogram of residuals and C) Q-Q plot of residuals.
(TIF)

Table S1 **Database of included regimens.** C = co-formulated; S = separate tablets; H = isoniazid; R = rifampin; E = ethambutol; Z = pyrazinamide; S = streptomycin; T = thiacetazone.
(PDF)
