# Supplementary figures and images for: Month 2 Culture Status and Treatment Duration as Predictors of Tuberculosis Relapse Risk in a Meta-Regression Model
Source: PLoS One. 2013 Aug 5;8(8):e71116. doi: 10.1371/journal.pone.0071116 (PMC3733776; doi:10.1371/journal.pone.0071116)

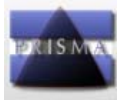

## PRISMA 2009 Flow Diagram

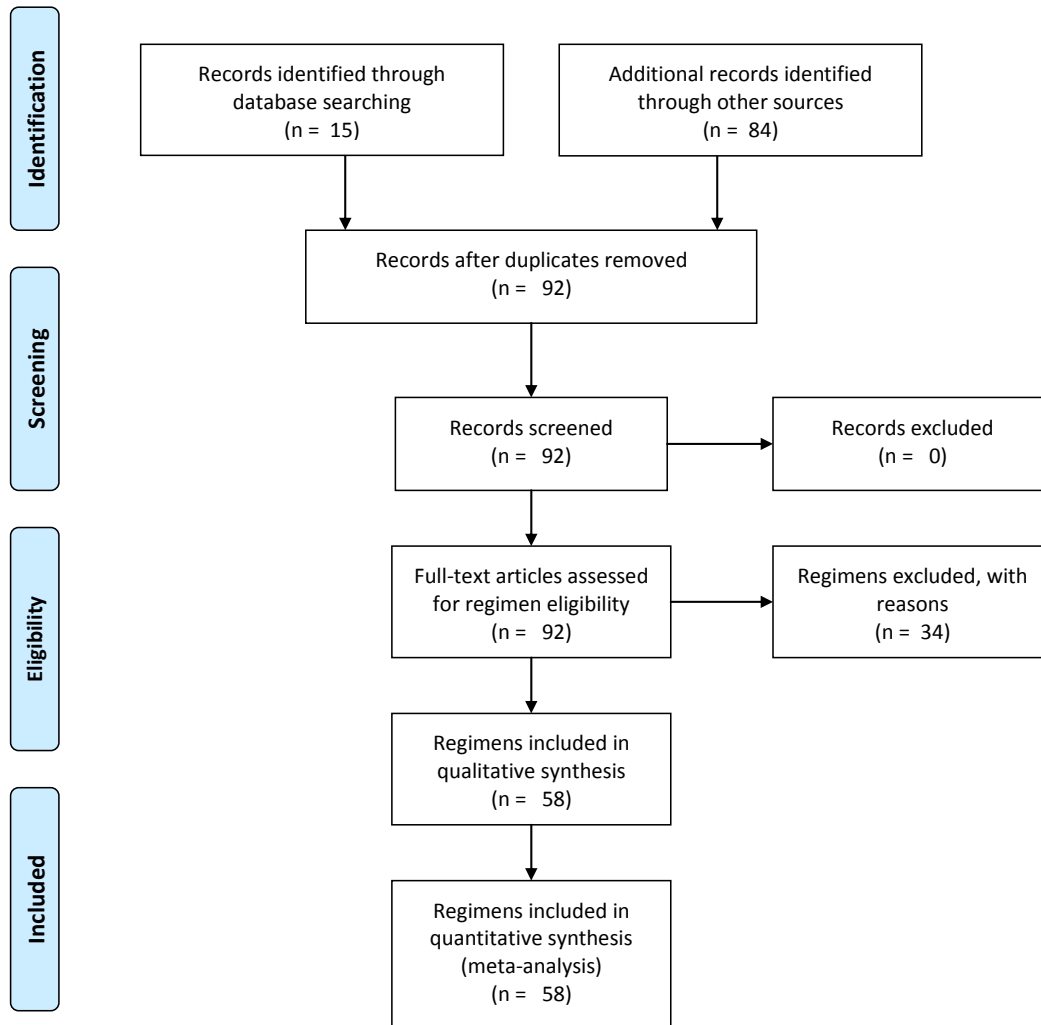

Supplement: Figure S1 — Study flow chart according to PRISMA guidelines. From reference [3] . (PDF) [file pone.0071116.s001.pdf]

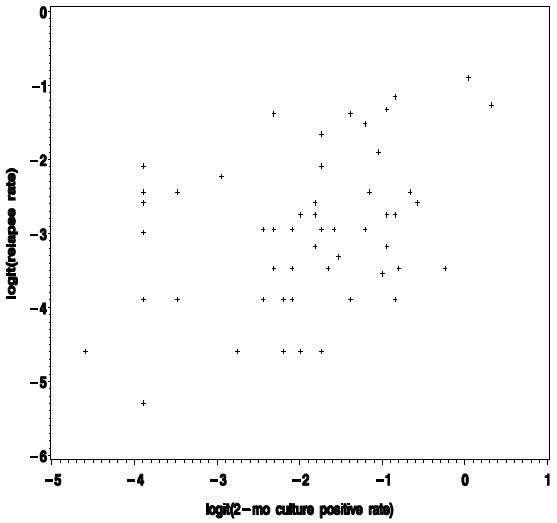

Supplement: Figure S3 — Scatter plot of logit 2-mo culture positive rates vs. logit relapse rates. (TIF) [file pone.0071116.s003.tif]

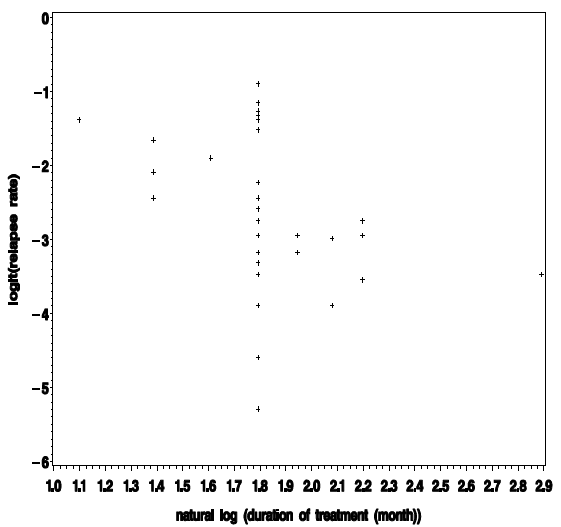

Supplement: Figure S4 — Scatter plot of natural log of treatment duration vs. logit relapse rates. (TIF) [file pone.0071116.s004.tif]

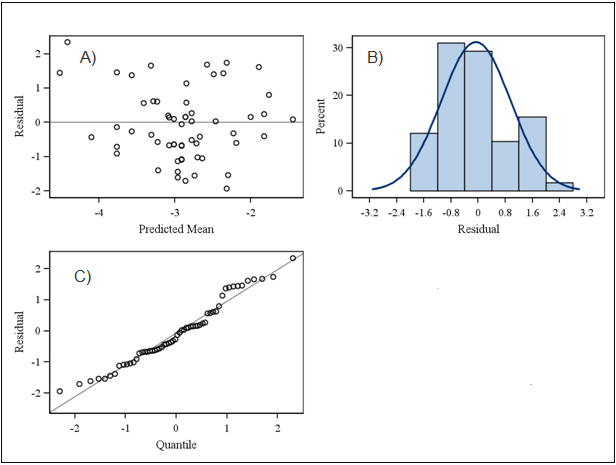

Supplement: Figure S5 — Diagnostic plots of standardized residuals for logit relapse rates. A) Predicted values vs. residuals, B) Histogram of residuals and C) Q-Q plot of residuals. (TIF) [file pone.0071116.s005.tif]
