# Supplementary material for: Month 2 Culture Status and Treatment Duration as Predictors of Tuberculosis Relapse Risk in a Meta-Regression Model
Source: PLoS One. 2013 Aug 5;8(8):e71116. doi: 10.1371/journal.pone.0071116 (PMC3733776; doi:10.1371/journal.pone.0071116)
Supplement: Table S1 — Database of included regimens. C = co-formulated; S = separate tablets; H = isoniazid; R = rifampin; E = ethambutol; Z = pyrazinamide; S = streptomycin; T = thiacetazone. (PDF) [file pone.0071116.s006.pdf]

Study no	Site	Regimen	Duration (mo)	Patients	Relapse risk	2-mo cult pos risk	RZ	Region	Refs
1	Africa	SHR	6	152	0.03	0.31	0.5	1	[1]
2	Africa	SHZ	6	153	0.08	0.34	0.5	1	[2]
3	Africa	SHT	6	104	0.22	0.58	0	1	[3]
4	Africa	2STH/TH	18	133	0.03	0.44	0	1	[4]
4	Africa	SH	6	112	0.29	0.51	0	1	[4]
5	Africa	SHR	6	171	0.02	0.3	0.5	1	[5]
6	Africa	HR	6	164	0.07	0.36	0.5	1	[6]
7	Africa	2SHRZ/HRZ	4	104	0.16	0.15	1	1	[7]
8	Africa	2SHRZ/HR	4	104	0.11	0.15	1	1	[8]
9	Africa	2SHRZ/HR	6	166	0.03	0.16	1	1	[9]
10	Africa	1.5SHRZ/H+(SRZ)	7	114	0.05	0.15	1	1	[10]
11	Hong Kong	$S_3H_3R_3Z_3$-Z_2*	6	220	0.03	0.09	0.5	0	[11]
11	Hong Kong	$S_3H_3R_3Z_3$-Z_6	6	208	0.03	0.09	0.5	0	[11]
11	Hong Kong	$S_3H_3R_3Z_3$-Z_4	6	205	0.05	0.09	0.5	0	[11]
11	Hong Kong	$H_3R_3Z_3$-Z_6	6	199	0.06	0.12	0.5	0	[11]
12	Hong Kong	$S_3H_3R_3Z_3$	6	49	0.2	0.2	0.5	0	[12]
12	Hong Kong	$S_3H_3R_3Z_3$	8	50	0.02	0.2	0.5	0	[12]
13	Singapore	2SHRZ(S)/H_3R_3	6	47	0	0.02	1	0	[13]
13	Singapore	1SHRZ(S)/H_3R_3	6	46	0.02	0.08	1	0	[13]
13	Singapore	2HRZ(S)/H_3R_3	6	44	0.02	0.03	1	0	[13]
13	Singapore	1SHRZ(C)/H_3R_3	6	42	0.05	0.08	1	0	[13]
13	Singapore	2SHRZ(C)/H_3R_3†	6	46	0.07	0.02	1	0	[13]
13	Singapore	2HRZ(C)/H_3R_3	6	40	0.08	0.03	1	0	[13]
14	India	$2S_3H_3R_3Z_3/S_2H_2R_2$	6	111	0.02	0.11	0.5	0	[14]
14	India	$2S_3H_3R_3Z_3/R_1H_1$	6	116	0.02	0.11	0.5	0	[14]
14	India	$2S_2H_2R_2Z_2/S_2H_2R_2$	6	108	0.03	0.11	0.5	0	[14]
14	India	$2S_3H_3R_3Z_3/R_2H_2$	6	101	0.03	0.11	0.5	0	[14]
14	India	$2S_2H_2R_2Z_2/S_1H_1R_1$	6	117	0.04	0.14	0.5	0	[14]
14	India	$2S_3H_3R_3Z_3/S_1H_1R_1$	6	111	0.05	0.11	0.5	0	[14]
14	India	$2S_2H_2R_2Z_2/R_2H_2$	6	102	0.06	0.14	0.5	0	[14]
14	India	$2S_2H_2R_2Z_2/R_1H_1$	6	109	0.07	0.14	0.5	0	[14]
15	Hong Kong	SHZ	9	65	0.05	0.23	0.5	0	[15,16]
15	Hong Kong	$S_3H_3Z_3$	9	65	0.06	0.3	0.5	0	[15,16]
15	Hong Kong	$S_2H_2Z_2$	9	49	0.06	0.28	0.5	0	[15,16]
15	Hong Kong	SHZ	6	60	0.18	0.23	0.5	0	[15,16]
15	Hong Kong	$S_2H_2Z_2$	6	39	0.21	0.28	0.5	0	[15,16]
15	Hong Kong	$S_3H_3Z_3$	6	68	0.24	0.3	0.5	0	[15,16]
16	Hong Kong	SHR	6	143	0.06	0.12	0.5	0	[17,18]
17	Singapore	2SHRZ/HRZ	6	78	0	0.02	1	0	[19–21]
17	Singapore	2SHRZ/HR	6	80	0.02	0.02	1	0	[19–21]
17	Singapore	2SHRZ/HR	4	77	0.08	0.02	1	0	[19–21]
17	Singapore	2SHRZ /HRZ	4	79	0.11	0.02	1	0	[19–21]
18	India	2SHZ/$S_2H_2Z_2$	7	129	0.04	0.28	0.5	0	[22,23]
19	India	SHRZ	3	200	0.2	0.09	1	0	[22,24]
19	India	3SHZ/$S_2H_2Z_2$	5	199	0.13	0.26	0.5	0	[22,24]
20	Hong Kong	$H_3R_3S_3Z_3E_3$	6	152	0.01	0.12	0.5	0	[25–27]
20	Hong Kong	$H_3R_3S_3Z_3$	6	151	0.01	0.1	0.5	0	[25–27]
20	Hong Kong	HRZE	6	163	0.01	0.06	1	0	[25,26,28]
20	Hong Kong	$H_3R_3Z_3E_3$	6	160	0.02	0.1	0.5	0	[25–27]
20	Hong Kong	$H_3R_3S_3E_3$	6	166	0.08	0.24	0.5	0	[25,26,28]
21	Singapore	2SHRZ/H_3R_3	6	97	0.01	0.01	1	0	[29,30]
21	Singapore	1SHRZ/H_3R_3	6	94	0.01	0.15	1	0	[29,30]
21	Singapore	2HRZ/H_3R_3	6	109	0.01	0.1	1	0	[29,30]
22	global	2HRE/7RH	9	445	0.028	0.269	0.5	0.5	[31]
22	global	2RHEZ/4RH	6	617	0.035	0.178	1	0.5	[31]
23	global	2RHEZ/4HR	6	347	0.05	0.17	1	0.5	[32]
24	Uganda	2HREZ/6HR	8	266	0.048	0.02	1	1	[33]
24	Uganda	2HREZ/4HR	6	147	0.097	0.05	1	1	[33]

References

1. East African-British Medical Research Councils (1972) Controlled clinical trial of short-course (6-month) regimens of chemotherapy for treatment of pulmonary tuberculosis. Lancet 1: 1079-1085.
2. East African-British Medical Research Councils (1973) Controlled clinical trial of four short-course (6-month) regimens of chemotherapy for treatment of pulmonary tuberculosis. Second report. Lancet 1: 1331-1338.
3. East African-British Medical Research Councils (1974) Controlled clinical trial of four short-course (6-month) regimens of chemotherapy for treatment of pulmonary tuberculosis. Third report. Lancet 2: 237-240.
4. East African-British Medical Research Councils (1977) Results at 5 years of a controlled comparison of a 6-month and a standard 18-month regimen of chemotherapy for pulmonary tuberculosis. Am Rev Respir Dis 116: 3-8.
5. East African-British Medical Research Councils (1974) Controlled clinical trial of four short-course (6-month) regimens of chemotherapy for treatment of pulmonary tuberculosis. Lancet 2: 1100-1106.
6. East African-British Medical Research Councils (1976) Controlled clinical trial of four 6-month regimens of chemotherapy for pulmonary tuberculosis. Second report. Am Rev Respir Dis 114: 471-475.
7. East African-British Medical Research Councils (1978) Controlled clinical trial of five short-course (4-month) chemotherapy regimens in pulmonary tuberculosis. First report of 4th study. Lancet 2: 334-338.
8. East African-British Medical Research Councils (1981) Controlled clinical trial of five short-course (4-month) chemotherapy regimens in pulmonary tuberculosis. Second report of the 4th study. Am Rev Respir Dis 123: 165-170.
9. East and Central African-British Medical Research Council (1983) Controlled clinical trial of 4 short-couse regimens of chemotherapy (three 6-month and one 8-month) for pulmonary tuberculosis. Tubercle 64: 153-166.
10. Tanzania-British Medical Research Council (1996) A controlled trial of a 4-weekly supplement of rifampicin, pyrazinamide and streptomycin in the continuation phase of a 7-month daily chemotherapy regimen for pulmonary tuberculosis. S Afr Med J 86: 960-965.
11. Hong Kong Chest Service-British Medical Research Council (1991) Controlled trial of 2, 4, and 6 months of pyrazinamide in 6-month, three-times-weekly regimens for smear-positive pulmonary tuberculosis, including an assessment of a combined preparation of isoniazid, rifampin, and pyrazinamide. Results at 30 months. Am Rev Respir Dis 143: 700-706.
12. Hong Kong Chest Service-Tuberculosis Research Centre-British Medical Research Council (1991) A controlled clinical comparison of 6 and 8 months of antituberculosis chemotherapy in the treatment of patients with silicotuberculosis in Hong Kong. Hong Kong Chest Service/tuberculosis Research Centre, Madras/British Medical Research Council. Am Rev Respir Dis 143: 262-267.
13. Singapore Tuberculosis Service-British Medical Research Council (1991) Assessment of a daily combined preparation of isoniazid, rifampin, and pyrazinamide in a controlled trial of three 6-month regimens for smear-positive pulmonary tuberculosis. Singapore Tuberculosis Service/British Medical Research Council. Am Rev Respir Dis 143: 707-712.
14. Balasubramanian R (1991) Fully intermittent six month regimens for pulmonary tuberculosis in south India. Indian J Tuberc 38: 51-53.
15. Hong Kong Tuberculosis Treatment Services-British Medical Research Council (1975) Controlled trial of 6- and 9-month regimens of daily and intermittent streptomycin plus isoniazid plus pyrazinamide for pulmonary tuberculosis in Hong Kong. Tubercle 56: 81-96.
16. Hong Kong Tuberculosis Treatment Services-British Medical Research Council (1977) Controlled trial of 6-month and 9-month regimens of daily and intermittent streptomycin plus isoniazid plus pyrazinamide for pulmonary tuberculosis in Hong Kong. The results up to 30 months. Am Rev Respir Dis 115: 727-735.

17. Hong Kong Chest Service-British Medical Research Council (1978) Controlled trial of 6-month and 8-month regimens in the treatment of pulmonary tuberculosis. First report. Am Rev Respir Dis 118: 219-228.
18. Hong Kong Chest Service-British Medical Research Council (1979) Controlled trial of 6-month and 8-month regimens in the treatment of pulmonary tuberculosis: the results up to 24 months. Tubercle 60: 201-210.
19. Singapore Tuberculosis Service-British Medical Research Council (1979) Clinical trial of six-month and four-month regimens of chemotherapy in the treatment of pulmonary tuberculosis. Am Rev Respir Dis 119: 579-585.
20. Singapore Tuberculosis Service-British Medical Research Council (1981) Clinical trial of six-month and four-month regimens of chemotherapy in the treatment of pulmonary tuberculosis: the results up to 30 months. Tubercle 62: 95-102.
21. Singapore Tuberculosis Service-British Medical Research Council (1986) Long-term follow-up of a clinical trial of six-month and four-month regimens of chemotherapy in the treatment of pulmonary tuberculosis. Am Rev Respir Dis 133: 779-783.
22. Santha T, Nazareth O, Krishnamurthy MS, Balasubramanian R, Vijayan VK et al. (1989) Treatment of pulmonary tuberculosis with short course chemotherapy in south India--5-year follow up. Tubercle 70: 229-234.
23. Tripathy SP, Ramakrishnan CV, Nazareth O, Parthasarathy R, Santha Devi T et al. (1983) Study of chemotherapy regimens of 5 and 7 months' duration and the role of corticosteroids in the treatment of sputum-positive patients with pulmonary tuberculosis in South India. Tubercle 64: 73-91.
24. Tuberculosis Research Centre MaNTIB (1986) A controlled clinical trial of 3- and 5-month regimens in the treatment of sputum-positive pulmonary tuberculosis in South India. Am Rev Respir Dis 134: 27-33.
25. Hong Kong Chest Service-British Medical Research Council (1982) Controlled trial of 4 three-times-weekly regimens and a daily regimen all given for 6 months for pulmonary tuberculosis. Second report: the results up to 24 months. Tubercle 63: 89-98.
26. Hong Kong Chest Service-British Medical Research Council (1981) Controlled trial of four thrice-weekly regimens and a daily regimen all given for 6 months for pulmonary tuberculosis. Lancet 1: 171-174.
27. Hong Kong Chest Service-British Medical Research Council (1987) Five-year follow-up of a controlled trial of five 6-month regimens of chemotherapy for pulmonary tuberculosis. Am Rev Respir Dis 136: 1339-1342.
28. Hong Kong Chest Service-British Medical Research Council (1987) Five-year follow-up of a controlled trial of five 6-month regimens of chemotherapy for pulmonary tuberculosis. Am Rev Respir Dis 136: 1339-1342.
29. Singapore Tuberculosis Service-British Medical Research Council (1988) Five-year follow-up of a clinical trial of three 6-month regimens of chemotherapy given intermittently in the continuation phase in the treatment of pulmonary tuberculosis. Am Rev Respir Dis 137: 1147-1150.
30. Singapore Tuberculosis Service-British Medical Research Council (1985) Clinical trial of three 6-month regimens of chemotherapy given intermittently in the continuation phase in the treatment of pulmonary tuberculosis. Am Rev Respir Dis 132: 374-378.
31. Combs DL, O'Brien RJ, Geiter LJ (1990) USPHS Tuberculosis Short-Course Chemotherapy Trial 21: effectiveness, toxicity, and acceptability. The report of final results. Ann Intern Med 112: 397-406.
32. Jindani A, Nunn AJ, Enarson DA (2004) Two 8-month regimens of chemotherapy for treatment of newly diagnosed pulmonary tuberculosis: international multicentre randomised trial. Lancet 364: 1244-1251.
33. Okwera A, Johnson JL, Luzze H, Nsubuga P, Kayanja H et al. (2006) Comparison of intermittent ethambutol with rifampicin-based regimens in HIV-infected adults with PTB, Kampala. Int J Tuberc Lung Dis 10: 39-44.
